# Aspirin has potential benefits for primary prevention of cardiovascular outcomes in diabetes: updated literature-based and individual participant data meta-analyses of randomized controlled trials

**DOI:** 10.1186/s12933-019-0875-4

**Published:** 2019-06-03

**Authors:** Samuel Seidu, Setor K. Kunutsor, Howard D. Sesso, J. M. Gaziano, J. E. Buring, Maria Carla Roncaglioni, Kamlesh Khunti

**Affiliations:** 1 Leicester Diabetes Centre, Leicester General Hospital, Gwendolen Road, Leicester, LE5 4WP UK; 2 Diabetes Research Centre, University of Leicester, Leicester General Hospital, Gwendolen Road, Leicester, LE5 4WP UK; 3 National Institute for Health Research Bristol Biomedical Research Centre, University Hospitals Bristol NHS Foundation Trust and University of Bristol, Bristol, UK; 4 Translational Health Sciences, Bristol Medical School, Musculoskeletal Research Unit, University of Bristol, Learning & Research Building (Level 1), Southmead Hospital, Bristol, BS10 5NB UK; 5 Division of Preventive Medicine, Brigham and Women’s Hospital, 900 Commonwealth Avenue, East-3rd Floor, Boston, MA 02215 USA; 6 Department of Medicine, Division of Aging, Brigham and Women’s Hospital, 75 Francis Street, Boston, MA 02115 USA; 7 Istituto di Ricerche Farmacologiche Mario Negri-IRCCS, Milan, Italy

**Keywords:** Aspirin, Diabetes, Primary prevention, Cardiovascular disease, All-cause mortality, Meta-analysis

## Abstract

**Background:**

The clinical benefit of aspirin for the primary prevention of cardiovascular disease (CVD) in diabetes remains uncertain. To evaluate the efficacy and safety of aspirin for the primary prevention of cardiovascular outcomes and all-cause mortality events in people with diabetes, we conducted an updated meta-analysis of published randomised controlled trials (RCTs) and a pooled analysis of individual participant data (IPD) from three trials.

**Methods:**

Randomised controlled trials of aspirin compared with placebo (or no treatment) in participants with diabetes with no known CVD were identified from MEDLINE, Embase, Cochrane Library, and manual search of bibliographies to January 2019. Relative risks with 95% confidence intervals were used as the summary measures of associations.

**Results:**

We included 12 RCTs based on 34,227 participants with a median treatment duration of 5.0 years. Comparing aspirin use with no aspirin, there was a significant reduction in risk of major adverse cardiovascular events (MACE)0.89 (0.83–0.95), with a number needed to treat (NNT)of 95 (95% CI 61 to 208) to prevent one MACE over 5 years average follow-up. Evidence was lacking of heterogeneity and publication bias among contributing trials for MACE. Aspirin use had no effect on other endpoints including all-cause mortality; however, there was a significant reduction in stroke for aspirin dosage ≤ 100 mg/day 0.75 (0.59–0.95). There were no significant effects of aspirin use on major bleeding and other bleeding events, though some of the estimates were imprecise. Pooled IPD from the three trials (2306 participants) showed no significant evidence of an effect of aspirin on any of the outcomes evaluated; however, aspirin reduced the risk of MACE in non-smokers 0.70 (0.51–0.96) with a NNT of 33 (95% CI 20 to 246) to prevent one MACE.

**Conclusions:**

Aspirin has potential benefits in cardiovascular primary prevention in diabetes. The use of low dose aspirin may need to be individualised and based on each individual’s baseline CVD and bleeding risk.

*Systematic review registration* PROSPERO: CRD42019122326

**Electronic supplementary material:**

The online version of this article (10.1186/s12933-019-0875-4) contains supplementary material, which is available to authorized users.

## Introduction

Cardiovascular disease (CVD)accounts for more than 70% of deaths in people with diabetes and is the leading cause of mortality in these patients [1]. Low-dose aspirin has commonly been used in the treatment and prevention of CVD and its effectiveness for the secondary prevention of CVD in people with diabetes is well established [2]. However, the balance of the benefits and harms of aspirin for primary CVD prevention in diabetes has been widely studied and still controversial. Following the Antithrombotic Treatment Trialists’ Collaboration 2009 meta-analysis of individual participant data (IPD) from six primary prevention trials which reported a non-significant reduction in serious vascular events in people with diabetes; [3] several other meta-analyses in people with diabetes have reported no significant benefit for aspirin in primary CVD prevention [4–7]. Recommendations from various guideline bodies on the use of aspirinin people with diabetes have not been consistent. Where as guidelines of the Fifth Joint Task Force of the European Society of Cardiology and Other Societies do not provide specific recommendations for the use of aspirin in people with diabetes [8], those by the American Diabetes Association (ADA), the American Heart Association (AHA), and the American College of Cardiology Foundation (ACCF) recommend the use of low-dose aspirin for the primary prevention of CVD in adults with diabetes, based on their individual baseline CVD risk and risk for bleeding [5]. In a recent updated meta-analysis of 10 trials published by our group [9], the data suggested a modest potential benefit of aspirin in the primary prevention of major adverse cardiovascular events (MACE) in people with diabetes; there was also an increase in the risk of major or gastrointestinal bleeding events, but estimates were imprecise and not significant. Our absolute risk reduction estimates and the potential for an increased risk of major bleeding events suggested that the benefits might not exceed the harms. Our recommendation was that further evidence was required.

In 2018, three separate landmark trials were published on role of aspirin in the primary prevention of CVD and these included (i) A Study of Cardiovascular Events in Diabetes (ASCEND); [10] (ii) Aspirin to Reduce Risk of Initial Vascular Events (ARRIVE); [11] and (iii) Aspirin in Reducing Events in the Elderly (ASPREE) [12]. The ASCEND and ASPREE trials assessed the effects of aspirin therapy in adults with diabetes mellitus exclusively and as a subgroup respectively. In the ASCEND trial, aspirin use reduced serious vascular events, but was associated with major bleeding events; whereas in the ASPREE trial, there was no differential effects of aspirin on the risk of CVD and bleeding in people with diabetes. Given the overall evidence, it appears the role of low-dose aspirin as a primary CVD prevention strategy in people with diabetes is still unresolved. Whether the potential benefits of aspirin for CVD primary prevention in diabetes is greater in those with high or low baseline CVD risk is also debated. Guidelines by the ADA recommend the use of low-dose aspirin for the primary prevention of CVD in adults who are at increased CVD risk, whilst not recommended for people at low CVD risk [13]. However, data from our previous review [9] and the ASPREE trial did not clearly support guidelines that encouraged the use of aspirin for the primary prevention of CVD in adults with diabetes at high CVD risk.

Given the persisting uncertainties on the benefits and harms of aspirin for the primary prevention of CVD in people with diabetes, we sought to update the evidence by conducting an updated meta-analysis of the literature. To enable comparison of the effectiveness of aspirin with placebo (or no treatment) under relevant clinical characteristics not collected by the literature-based meta-analysis, we also conducted an (Individual Patient Data) IPD meta-analysis of three trials that were able to share their data.

## Methods

### Data sources and search strategy

We conducted this literature-based review and IPD using an updated protocol registered in the PROSPERO International prospective register of systematic reviews (CRD42019122326) and in accordance with guidelines of PRISMA (Additional file 1: Appendix S1) [14], PRISMA-IPD (Additional file 1: Appendix S2) [15], methods recommended by the IPD Meta-analysis Methods Group of the Cochrane Collaboration [16], and guidance of Riley and colleagues [17]. We searched MEDLINE, Embase, and The Cochrane Library for randomised controlled trials (RCTs) published from November, 2015 (date of the last search of our previous review [9]) to 29 January 2019. The computer-based searches combined free text and medical subject headings and combination of key words related to aspirin and diabetes, with no restrictions placed on language. The full details of the search strategy are provided in Additional file 1: Appendix S3. The titles and abstracts of all articles identified by the literature search were initially screened to assess their suitability for inclusion, after which we acquired potentially relevant articles for detailed full text evaluation. Two reviewers (SKK and SS) independently conducted full text evaluation using the inclusion criteria and any disagreements regarding eligibility of an article was discussed. We also searched reference lists of selected studies and relevant reviews for additional publications. No separate ethical approval was required for the conduct of this study, as any necessary ethical approval was obtained for each of the individual studies contributing data to the meta-analysis.

### Study selection and eligibility criteria

Intervention studies were eligible if they met the following inclusion criteria: (i) were randomised controlled, open or blinded trials; (ii) assessed the effects of aspirin therapy compared to a placebo or no treatment; (iii) enrolled adults with diabetes mellitus (either exclusively or as a subgroup) without previous history or clinical evidence of CVD; (iv) reported data on cardiovascular endpoints, all-cause mortality, or other adverse events such as bleeding, cancer etc.; and (v) and had a follow-up duration of at least a year. Studies that were non-randomised comparing aspirin with another antiplatelet agent, included people with known CVD, or were secondary publications of trials already included in the analysis were excluded from the review.

### Data extraction and quality assessment

One author (SKK) initially conducted the data extraction using a standardized data collection form and a second author (SS) independently checked the extracted data with that in the original articles. Data were extracted on the following characteristics: study design; patient characteristics, intervention and comparison, and outcomes. The risk of bias assessment was conducted using the Cochrane Collaboration’s risk of bias tool [18].

### Outcomes

The primary outcomes were MACE) [defined as composite of nonfatal myocardial infarction (MI), nonfatal stroke, and cardiovascular death] and all-cause mortality. Secondary outcomes included (i) other cardiovascular endpoints (nonfatal MI, coronary heart disease death, fatal and nonfatal stroke, revascularization, sudden coronary death, and transient ischaemic attack) and adverse events (any bleeding, gastro-intestinal bleeding, cancer, allergic reactions, and arrhythmias). Cardiovascular outcomes used in trials and their ascertainment are reported in Additional file 1: Appendix S4.

### Aspirin in Primary Prevention of cArdiovasculardIseaSe in diabEtes (APPRAISE) IPD meta-analysis consortium

Investigators of eligible trials identified by the literature search strategy and well-known investigators in the field, were contacted by email, letter or phone, provided with a summary of the study protocol, and invited to join the collaboration if they had the relevant data available. Investigators expressing interest to collaborate in this effort were then provided with full details of the study protocol and a list of relevant study variables that could be used in the analyses. Data from each study were obtained using a standardised spreadsheet or in STATA format. The raw data were examined and inconsistencies or irregularities were clarified with the investigators. Individual level data collected was coded and entered into a single database.

### Statistical analysis

For the literature-based meta-analysis, summary measures were presented as relative risks (RRs) with 95% confidence intervals (CIs). Following Cornfield’s rare disease assumption [19], reported hazard ratios (HRs) and odds ratios (ORs) were assumed to approximate the same measure of RRs. We used reported RRs or calculated study specific unadjusted RRs based on raw events. To minimize the effect of heterogeneity, random-effects models was used to pool RRs. Heterogeneity across studies was assessed using the Cochrane *χ*^*2*^ statistic and the *I*^*2*^ statistic [20]. Study level characteristics including geographical location, sex differences, allocation concealment, baseline CVD risk, dose of aspirin, compliance, duration of treatment, and number of outcomes were prespecified as characteristics for assessment of heterogeneity, which were evaluated using stratified analysis and random effects meta-regression. For comparisons involving 10 or more studies, publication bias was assessed by visually inspecting a funnel plot and applying Egger’s regression symmetry test [21].

In the IPD meta-analysis, since not all trials provided time to event data, effects of the intervention on outcomes were expressed as ORs using logistic regression analysis in the main analysis. Cox proportional hazards models were used for time to event data in supplementary analyses. We employed a one-step IPD meta-analyses in which IPD from all studies were modelled simultaneously with fixed effects, adjusting for age and trial arm to obtain the pooled intervention effect with a 95% CI. To contextualise our results, we also calculated the number needed to treat (NNT) using the formula: NNT = 1/absolute risk reduction (ARR). The ARR was derived by multiplying the control risk by the relative risk reduction. All statistical analyses were performed with Stata release 15 (StataCorp, College Station, Texas, USA).

## Results

### Literature-based meta-analysis

#### Study identification and selection

**Fig. 1:**
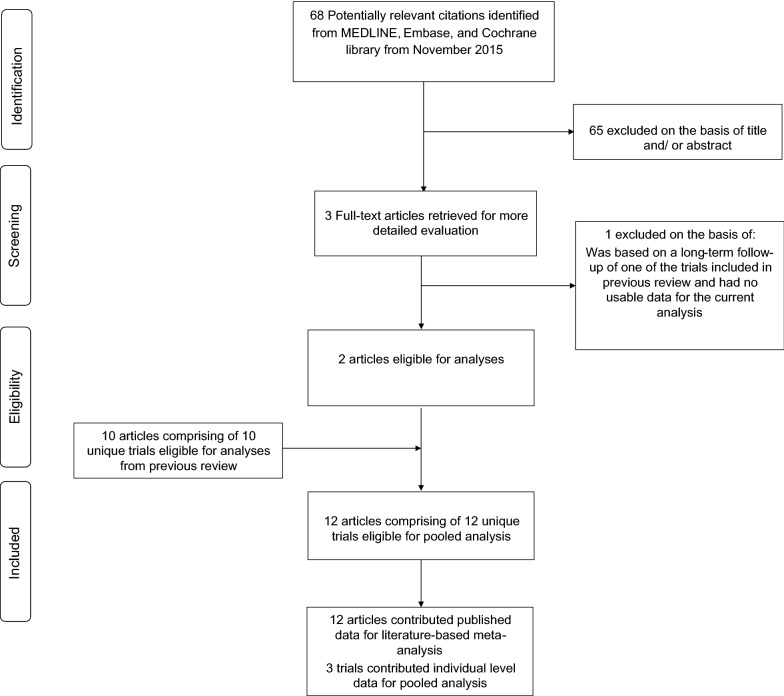
PRISMA flow diagram

The systematic search of databases and manual scanning of reference lists from November 2015 to January 2019 identified 68 potentially relevant articles. Following screening based on titles and abstracts, three articles remained for further evaluation. Following detailed full text evaluation, one was excluded. The remaining two articles plus 10 articles identified from our previous review yielded a total of 12 unique trials for the meta-analysis [10, 12, 22–31], which in aggregate comprised of 34,227 participants with diabetes (Fig. 1).

#### Study characteristics and quality

Key characteristics of the 12 eligible trials published between 1988 and 2018 are reported in Table 1. Eight trials were double-blinded and the remaining were open label trials. The trials were conducted in different geographical locations: Five in Europe (UK and Italy); three in North America (USA); two in Asia (Japan); one recruited participants from both USA and Australia and another from 26 countries in Europe, North and South America, and Asia. The baseline average age of trial participants ranged from 18 to 90 years. There was variability in study populations ranging from participants at low to high cardiovascular risk: healthy community dwelling participants, those with pre-existing conditions such as hypertension, and those with multiple atherosclerotic risk factors. Only four trials were conducted specifically in people with diabetes and the remaining eight were based on data from subgroup analysis in people with diabetes. The Early Treatment Diabetic Retinopathy Study (ETDRS) trial made a distinction between type 1 and type 2 diabetes in their results and also included a small proportion of people with pre-existing CVD [24]. In the ASCEND trial, over 94% of study participants were reported to have type 2 diabetes [10]. The dosage of aspirin ranged from 75 mg to 650 mg daily. Medication compliance or adherence was reported in seven trials using a variety of subjective (self-reports) and objective (biochemical monitoring and pill counts) measures and average values ranged from 50.0 to 91.8%. The ASPREE trial reported that approximately two-thirds of participants were still taking their assigned intervention at the end of the trial [12]. The average follow-up times of included trials ranged from 3.6 to 10.1 years and the overall median (interquartile range) follow-up time was 5.0 (4.5–6.7) years. Eight trials demonstrated a high risk of bias within one to three areas of study quality, as assessed using the Cochrane Collaboration tool (Additional file 1: Appendix S5). Majority of the trials had a high risk of bias for selective reporting. Only two trials were found to have a low risk of bias in all areas and eight trials had an unclear risk of bias in one or more areas of study quality.

**Table 1:** Characteristics of clinical trials of aspirin therapy included in meta-analysis

Lead Author, publication date	Name of study or source of participants	Study design	Patient population	Location	Baseline year of study	Age group	Males (%)	Allocation concealment	Blinding to subjects	Blinding to carers	Aspirin dose	Medication compliance (%)	Duration of therapy (years)	Completeness of follow-up	Trial participants with diabetes
Peto, 1988	BMD	Randomised, open label with no placebo	Healthy male doctors	UK	1978–1979	19–90	100.0	No	No	No	500 mg daily	NR	5.6	Unclear	101
PHS Steering Committee, 1989	PHS	RCT, double blinded	Healthy male doctors	USA	1982	40–84	100.0	Unclear	Yes	Yes	325 mg every other day	NR	5.0	99.7	533
ETDRS Investigators, 1992	ETDRS	RCT, double blinded	Participants with type 1 and 2 diabetes	USA	1980–1985	18–70	56.5	Unclear	Yes	Yes	650 mg daily	91.8	5.0	94.7	3711
MRC, 1998	TPT	Randomized, placebo controlled. Factorial with initial parallel group phase	Patients at high risk for IHD	UK	1989–1994	45–69	100.0	Adequate	Yes	Yes	75 mg daily	NR	6.7	98.9	68
Hansson, 1998	HOT	RCT, double blinded	Participants with hypertension	Multiple countries	1992–1994	50–80	NR	Adequate	Yes	Yes	75 mg daily	NR	3.8	97.4	1501
Sacco, 2003	PPP	Randomised open trial with 2 × 2 factorial design	Participants > 50 years with one or more CV risk factors	Italy	NR	64.3*	48.2	Adequate	No	No	100 mg daily	71.8	3.6	99.3	1031
Ridker, 2005	WHS	RCT, double blinded, 2 x 2 factorial	Healthy female health professionals	USA	1993	≥ 45	0.0	Unclear	Yes	Yes	100 mg on alternate days	NR	10.1	99.4	1027
Belch, 2008	POPADAD	RCT, double blinded, 2 x 2 factorial	Patients ≥ 40 years with type 1 and 2 diabetes, ABP <=0.99	Scotland	NR	≥ 40	44.1	Adequate	Yes	Yes	100 mg daily	50.0	6.7	99.5	1276
Ogawa, 2008	JPAD	Randomised open label with blinded end point assessment	Patients with type 2 diabetes	Japan	2002	65.0*	55.0	Adequate	No	No	81 or 100 mg daily	90.0	4.4	92.4	2539
Ikeda, 2014	JPPP	Randomised open label, parallel group	Elderly with multiple atherosclerotic risk factors	Japan	2005–2007	60–85	NR	Adequate	No	No	100 mg daily	76.0	5.0	~ 98.7	4903
McNeil, 2018	ASPREE	RCT, double blind	Community dwelling free of CVD, disability, dementia	USA, Australia	2010–2014	≥ 65	74.0	Adequate	Yes	Yes	100 mg daily	70.0	4.7	98.5	2057
ASCEND Study Group, 2018	ASCEND	RCT, double blind	Patients identified from diabetes registers or general practices	UK	2005–2017	≥ 40	63.0	Adequate	Yes	Yes	100 mg daily	70.0	7.4	99.1	15,480

ASCEND, A Study of Cardiovascular Events in Diabetes; ASPREE, Aspirin to Reduce Risk of Initial Vascular Events; BMD, British male doctors; ETDRS, Early Treatment Diabetic Retinopathy Study; HOT, Hypertension Optimal Treatment; IHD, ischaemic heart disease; JPAD, Japanese Primary Prevention of Atherosclerosis with Aspirin for Diabetes; JPPP, Japanese Primary Prevention Project; MRC, Medical Research Council; NR, not reported; PHS, Physicians’ Health Study; POPADAD, Prevention Of Progression of Arterial Disease And Diabetes; PPP, Primary Prevention Project; RCT, randomised controlled trial; UK, United Kingdom; USA, United States of America; WHS, Women’s Health Study

* Average age

### Major cardiovascular outcomes and all-cause mortality

**Fig. 2:**
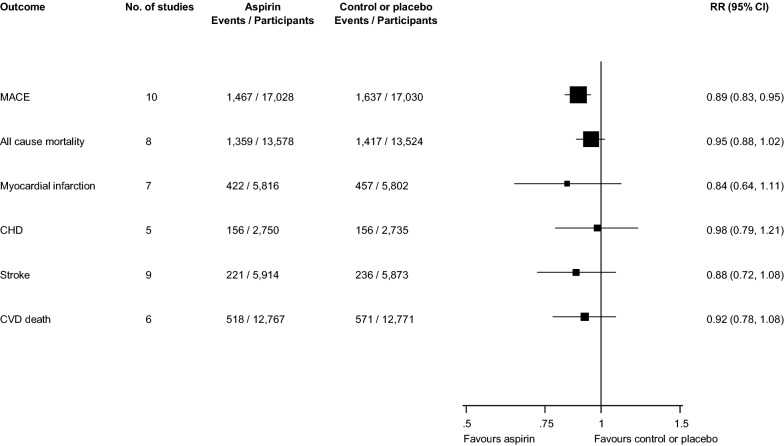
Effect of aspirin on the primary prevention of major adverse cardiovascular events, all-cause mortality, myocardial infarction, coronary heart disease, stroke, and cardiovascular disease death, in people with diabetes. CI, confidence interval (bars); CHD, coronary heart disease; CVD, cardiovascular disease; MACE, major adverse cardiovascular events; RR, relative risk

Relative risks for cardiovascular outcomes and all-cause mortality events comparing aspirin therapy with placebo or no treatment in pooled analyses are reported in Fig. 2 and Appendices S6–S12. In 10 trials of MACE (34,058 participants and 3104 events), aspirin therapy significantly reduced the risk of MACE compared with placebo or no treatment 0.89 (95% CI 0.83 to 0.95). Using a fixed effects model, the pooled RR remained unchanged (Additional file 1: Appendix S6). There was no evidence of heterogeneity between the contributing studies (*I*^*2*^= 0%, 0 to 62%; *p *> 0.99). On exclusion of the ETDRS trial which involved a small proportion of patients with previous CVD, the pooled RR remained the same 0.89 (95% CI 0.82 to 0.96) (Additional file 1: Appendix S7).

Aspirin therapy was not associated with a significant decrease in risk of all-cause mortality (eight trials; 27,102 participants and 2776 events) 0.95 (95% CI 0.88 to 1.02) and there was no evidence of heterogeneity between contributing studies (*I*^*2*^= 0%, 0 to 68%; *p *= 0.88) (Fig. 2; Additional file 1: Appendix S8).

In seven trials of MI and five trials of CHD, aspirin therapy was not associated with a significant reduction in risk of any of these outcomes: 0.84 (95% CI 0.64 to 1.11)and 0.98 (95% CI 0.79 to 1.21) respectively (Fig. 2; Additional file 1: Appendices S9, S10). There was evidence of moderate heterogeneity (*I*^*2*^= 57%, 1 to 82%; *p *= 0.03) for the MI analysis and no evidence of heterogeneity (*I*^*2*^= 0%, 0 to 79%; *p *= 0.75) for the CHD analysis.

There was no significant reduction in the risk of stroke or CVD death when aspirin therapy was compared with placebo or no treatment: 0.88 (95% CI 0.72 to 1.08) and 0.92 (95% CI 0.78 to 1.08;) respectively (Fig. 2; Additional file 1: Appendices S11, S12). There was no significant evidence of heterogeneity between the contributing studies for stroke or CVD death: (*I*^*2*^= 10%, 0 to 68%; *p *= 0.35) and (*I*^*2*^= 23%, 0 to 67%; *p *= 0.26) respectively.

### Other cardiovascular outcomes

**Fig. 3:**
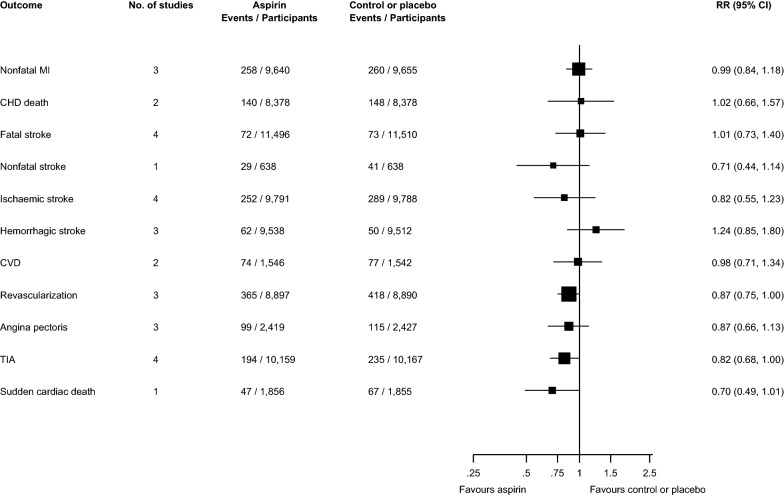
Effect of aspirin on the primary prevention of individual cardiovascular disease endpoints in people with diabetes. CHD, coronary heart disease; CI, confidence interval (bars); CVD, cardiovascular disease; MI, myocardial infarction; RR, relative risk; TIA, transient ischaemic attack

There was no significant difference in the risk of other cardiovascular outcomes such as nonfatal MI, CHD death, fatal stroke, nonfatal stroke, ischaemic stroke, haemorrhagic stroke, CVD, revascularization, angina pectoris, sudden coronary death, and TIA, when aspirin was compared with placebo or no treatment (Fig. 3; Additional file 1: Appendix S13).

### Subgroup analysis

There was no evidence of effect modification by any of the clinically relevant study-level characteristics explored for the outcomes of MACE, all-cause mortality, and CVD death (Additional file 1: Appendices S14–S18). For MI, the moderate heterogeneity between studies contributing to pooled analysis seemed to be partly explained by treatment duration (*p* value for meta-regression = 0.01). Compared to participants with treatment duration more than 5 years, participants with treatment duration of 5 years or less had a significantly reduced risk of MI with aspirin use 0.70 (95% CI 0.53 to 0.93) (Additional file 1: Appendix S16). There was also evidence of effect modification by aspirin dosage (*p* value for meta-regression = 0.02) and treatment duration (*p* value for meta-regression = 0.02) for the effect of aspirin use on stroke. The risk of stroke was significantly reduced for trials using aspirin dosage of 100 mg per day or less compared to more than 100 mg per day. Similarly, compared to participants with treatment duration of 5 years or less, participants with treatment duration of more than 5 years had a significantly reduced risk of stroke with aspirin (Additional file 1: Appendix S17).

### Adverse effects

**Fig. 4:**
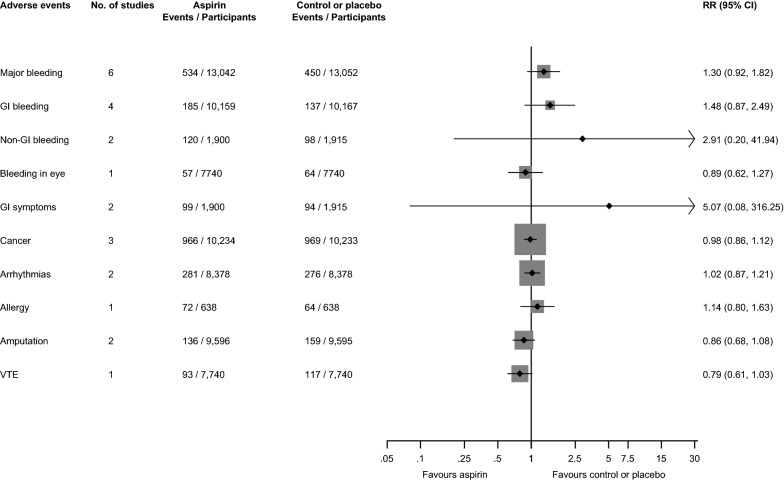
Effect of aspirin on adverse events in people with diabetes. CI, confidence interval (bars); GI, gastro-intestinal; RR, relative risk; VTE, venous thromboembolism

Figure 4 presents pooled RRs of the effects of aspirin therapy compared with placebo or no treatment on adverse outcomes such as major bleeding, gastrointestinal bleeding, non-gastrointestinal bleeding, cancer, venous thromboembolism, gastrointestinal symptoms, arrhythmias, and allergy. No statistically significant difference in risk was found for any of these outcomes.

### Absolute benefits and harms

The ARR of MACEin people with diabetes associated with aspirin use was 1.06% (95% CI 0.48 to 1.63)over the average follow-up period of 5 years, which translates into a NNT of 95 (95% CI 61 to 208) to prevent one MACE. For MI in participants with a treatment duration of 5 years or less, the ARR and NNT were 2.26 (95% CI 0.53 to 3.55) and 44 (95% CI 28 to 189) respectively. For the risk of stroke with aspirin dosage of 100 mg per day or less, the ARR and NNT were 0.99 (95% CI 0.20 to 1.62) and 101 (95% CI 62 to 507) respectively. For the risk of stroke with treatment duration of more than 5 years, the ARR and NNT were 2.41 (95% CI 0.62 to 3.64) and 42 (95% CI 27 to 172) respectively.

### Publication bias

A funnel plot for the comparison that involved 10 studies (MACE) was symmetrical (Additional file 1: Appendix S19) which was consistent with Egger’s regression symmetry test (*p *= 0.99). In addition, there was no definitive evidence of selective reporting when studies were grouped by size in meta-regression analysis (Additional file 1: Appendix S14).

### Analysis of IPD of RCTs

**Fig. 5:**
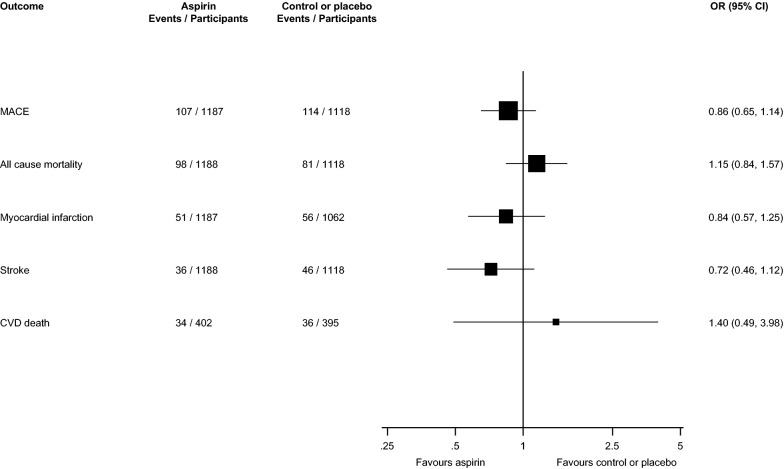
Effect of aspirin on the primary prevention of major adverse cardiovascular events, all-cause mortality, myocardial infarction, stroke, and cardiovascular disease death in people with diabetes, based on pooled analysis of individual level data from three trials. CI, confidence interval (bars); CVD, cardiovascular disease; MACE, major adverse cardiovascular events; OR, odds ratio. BP, blood pressure; CI, confidence interval (bars); OR, odds ratio; RA, rheumatoid arthritis

**Fig. 6:**
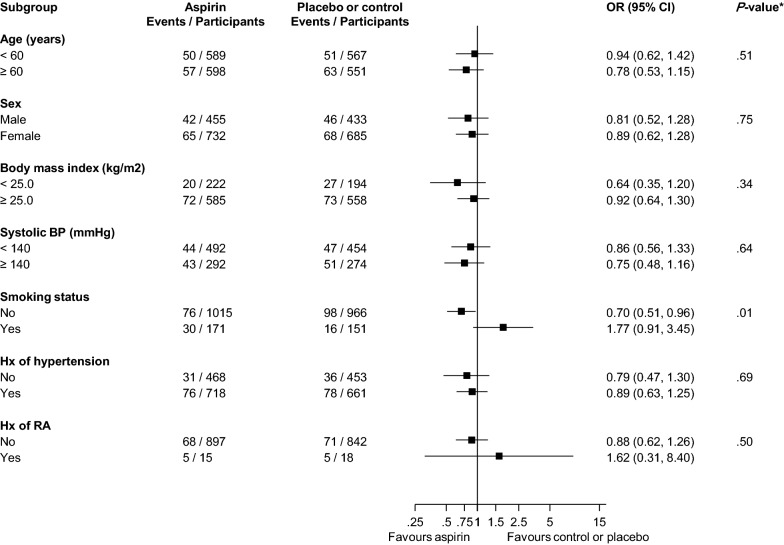
Effect of aspirin on the primary prevention of major adverse cardiovascular events according to relevant clinical characteristics, based on pooled analysis of individual level data from three trials. BP, blood pressure; CI, confidence interval (bars); OR, odds ratio; RA, rheumatoid arthritis

Investigators of 10 trials were contacted to contribute data to the APPRAISE consortium. Of these 10, we obtained IPD data from three RCTs namely: Physicians’ Health Study (PHS); Primary Prevention Project (PPP); and the Women’s Health Study (WHS). Details of contributing trials are presented in Additional file 1: Appendix S20. The three RCTs comprised of 2306 participants of which 1188 were randomised to the aspirin group and 1118 to the placebo group (Table 2). Overall the mean (standard deviation, SD) age was 60.1 (8.7) years and 38.5% were male. The median (interquartile range) duration of follow-up was 9.5 (5.2–10.4) was years (based on data from two trials). The trial groups had similar cardiovascular risk profiles. In pooled analysis of the three trials, there was no significant evidence of an effect of aspirin on MACE, all-cause mortality, MI, stroke, or CVD death (Fig. 5). In pooled analysis of two trials (PHS and WHS) with time to event data, there was no significant difference in risk for any of the outcomes assessed (Additional file 1: Appendix S21). There was significant evidence of a differential effect of aspirin on the risk of MACE by smoking status ((*p* value for meta-regression = 0.01) (Fig. 6). Aspirin use reduced the risk of MACE in non-smokers 0.70 (95% CI 0.51 to 0.96) compared to those who smoked 1.77 (95% CI 0.91 to 3.45). The ARR of MACE in non-smokers associated with aspirin use was 3.04% (95% CI 0.41 to 4.97) over the average follow-up period of 10 years, which translates into a NNT of 33 (95% CI 20 to 246) to prevent one MACE.

**Table 2:** Key characteristics of participants at baseline in three trials that contributed individual participant data

Characteristic	Aspirin group (N = 1188)	Placebo group (N = 1118)
Age (years)	60.1 (8.9)	60.1 (8.6)
Male sex	455 (38.3)	433 (38.7)
Body mass index (kg/m^2^)	28.8 (5.7)	29.1 (6.0)
Systolic blood pressure (mmHg)	134.8 (14.4)	134.7 (13.8)
Total cholesterol (mmol/l)*	5.66 (1.20)	5.41 (1.16)
Smoking status		
Other	1015 (85.5)	966 (86.5)
Current	172 (14.5)	151 (13.5)
History of hypertension		
No	469 (39.5)	453 (40.7)
Yes	718 (60.5)	661 (59.3)
Baseline treatment for hypertension*		
No	489 (61.4)	455 (61.6)
Yes	307 (38.6)	284 (38.4)
Family history of MI*		
No	666 (84.0)	623 (84.2)
Yes	127 (16.0)	117 (15.8)
History of RA		
No	898 (98.4)	842 (97.9)
Yes	15 (1.6)	18 (2.1)

* Data contributed by two trials; values are mean (SD), median (IQR), or n (%); MI, myocardial infarction; rheumatoid arthritis

## Discussion

### Key findings

Using a literature-based meta-analysis and pooled analysis of individual level data from the PHS, PPP, and WHS trials, we have evaluated the efficacy and safety of aspirin for the primary prevention of cardiovascular outcomes and all-cause mortality events among people with diabetes. Compared with no aspirin, aspirin use was associated with an 11% reduction in the risk of MACE (which was a combination of cardiovascular mortality, nonfatal MI, and nonfatal stroke outcomes). Aspirin use however no effect on other cardiovascular endpoints and all-cause mortality. Heterogeneity was generally low or absent between contributing studies for majority of outcomes, except for the MI comparison which was characterised by moderate heterogeneity. Except for MI and stroke, there were no differential effects of aspirin on other outcomes by the pre-specified clinically relevant characteristics including baseline cardiovascular risk. Aspirin use was associated with a 30% reduction in the risk of MI for a treatment duration of 5 years or less, but no benefit for a treatment duration of more than 5 years. In addition, aspirin use reduced the risk of stroke for a lower intervention dose (≤ 100 mg/day) and longer treatment duration (> 5 years). In the context of multiple statistical tests for interaction conducted, the results of the subgroup analyses should be interpreted with caution and may require replication in further studies. There were suggestions that aspirin use might be associated with adverse effects such as major bleeding, gastrointestinal symptoms, and arrhythmias, but the estimates were imprecise and not significant. The effects of aspirin on cancer and venous thromboembolism outcomes were also not significant. Finally, pooled analysis of individual level data from three trials showed no significant evidence of an effect of aspirin on any of the outcomes evaluated. However, aspirin use was associated with a 30% reduction in the risk of MACE in non-smokers; with a suggestion of an increased risk of MACE in smokers, though the risk estimate in this group was based on only 46 events and was imprecise and should be interpreted with caution. These findings may reflect evidence that cigarette smoking causes an increase in platelet aggregability [32, 33], hence aspirin may not effectively inhibit platelet activity in smokers. It has been reported that smokers may be about 12 times resistant to the effects of aspirin. An established body of literature suggests that the reduced antiplatelet effect of aspirin is associated with a fourfold increase in the risk of adverse cardiovascular events [34, 35].

### Comparison with previous work

The current study builds on previous meta-analyses including one of ours [4–7, 9]. Since the publication of the ASCEND-A Study of Cardiovascular Events in Diabetes and ASPREE-Aspirin in Reducing Events in the Elderly trials in 2018, a number of reviews have recently been published. In one review which was based on the overall population and not specifically in people with diabetes [36], Zheng and Roddick in a subsidiary analysis reported data for participants with diabetes based on 10 studies. In their report, aspirin therapy was associated with a reduction in the primary composite cardiovascular outcome (MACE) based on pooled analysis of seven trials, but not with any of the secondary cardiovascular outcomes. In addition, they reported increases in major and gastrointestinal intestinal bleeding. In a research letter, Fortuni and colleagues included only five trials in their pooled analysis and reported similar findings to that of Zheng and Roddick [37]. Our current study which is based on pooled analysis of 12 trials is therefore an updated assessment of the topic and also includes a pooled analysis of IPD contributed by three trials. Consistent with the recent and other previously published reviews on the topic [4–7, 9, 36], aspirin use reduced MACE with no differential effects of aspirin use on other cardiovascular endpoints as well as all-cause mortality. New findings by our current study include important differences by aspirin dosage and treatment duration for the effect of aspirin use on the risk of MI and stroke and no effects of aspirin use on major bleeding and other adverse effects. Based on IPD contributed by three trials, we had the opportunity to re-evaluate the efficacy of aspirin for the specified outcomes in clinically relevant subgroups with suggestions of an important difference by smoking status for the effect of aspirin on the risk of MACE.

### Implications of our findings

Compared with aspirin use for the secondary prevention of vascular disease, the use of aspirin for primary prevention has been a widely debated and controversial topic. Two recently published landmark trials, ASCEND, and ASPREE, [10, 11] evaluated the efficacy and safety of aspirin for the primary prevention of cardiovascular outcomes in people with diabetes and have been included in this review. Whereas the absolute benefits of aspirin on vascular benefits were counterbalanced by the bleeding hazard in the ASCEND study, there was no differential effect of aspirin on the risk of CVD and bleeding in people with diabetes in the ASPREE trial. Though our results show aspirin may have a beneficial effect in the primary prevention of cardiovascular disease in people with diabetes, aspirin had no differential effect on bleeding risk. Given the imprecise estimates reported for GI and non-GI bleeding, these results may be due to inadequate power of these trials to detect these events. Indeed, a wealth of data from general and secondary prevention populations suggests that the main adverse effect associated with aspirin use is GI bleeding [38]. Both low aspirin dosage and long term therapy is associated with an absolute excess of GI bleeding complications in these population groups [39]. A higher risk of bleeding events has also been reported among the elderly and people at low cardiovascular risk [40]. Though the current data suggests otherwise, real-world data in general populations have demonstrated higher rates of bleeding in people with diabetes on aspirin therapy [41]. Taking the overall findings together, two questions still remain on the role of aspirin therapy in primary prevention of CVD in diabetes: (i) are the absolute vascular benefits of aspirin counterbalanced by the potential for bleeding and (ii) in what groups of the population do the benefits of aspirin outweigh its bleeding hazards. Based on a recent comprehensive narrative review by Lippi and colleagues, the authors suggest that the harms of aspirin in primary prevention of CVD may be larger than the benefits, especially in the elderly general population [42]. Our study findings suggest there may be important differences in the effect of aspirin by treatment dosage, treatment duration, as well as smoking status, but these results are based on limited data from subgroup analyses. Though our findings showed no suggestions of differences in the effect of aspirin by gender on all outcomes evaluated, there is evidence suggesting that the variation in the effect of aspirin therapy on cardiovascular outcomes could be explained by gender. In a meta-analysis of 23 trials which aimed to evaluate whether gender might play a role in explaining the large variation of aspirin efficacy across primary and secondary MI prevention trials, the authors demonstrated that gender accounted for a substantial proportion of the variability in the efficacy of aspirin in reducing MI rates across these trials [43]. Emerging evidence points to the fact that women have an increased risk of aspirin resistance compared to men, which makes aspirin less effective in women [44]. Apart from the condition diabetes which is associated with reduced rates of responsiveness to aspirin [45], other factors that could potentially reduce the antiplatelet effect of aspirin include older age, obesity, renal insufficiency, and platelet count. To answer all these pertinent questions may require an IPD meta-analysis based on all contributing trials. An updated meta-analysis by the Antithrombotic Trialists’ Collaboration could help address some of these questions. There have been contradictory guideline recommendations on the role of aspirin in primary CVD prevention; however, the ADA recommends the use of low-dose aspirin for the primary prevention of CVD in adults with type 1 and 2 diabetes who are at increased CVD risk, whereas not recommended for people at low CVD risk as the potential for bleeds likely offsets potential benefits in these people [13]. Though the current findings do not justify these recommendations, it appears the clinical decision to initiate low dose aspirin for primary prevention is a complex process and should be individualised and tailored to each patient’s baseline CVD and bleeding risk, as these tend to differ from one individual to another. Patients preferences should also be taken into account when making the decision. Leggio and colleagues in their review recommend that when a decision is taken to initiate primary CVD prevention, uncoated aspirin formulations with higher bioavailability should be prescribed at the lowest effective lower dose and concurrently used with proton-pump inhibitors for those at high risk of gastrointestinal bleeding [46]. In addition, concurrent use of nonsteroidal anti-inflammatory drugs should be avoided and those with rapid platelet turnover should be considered for twice-daily dosing regimen [46].

### Strengths and limitations

Several strengths of this review deserve consideration. Compared to previous reviews including the most recent one [36], it is the largest and most comprehensive review on the topic to date. In total we included 12 trials with additional data contributed by three trials that shared their individual level data, hence we had the ability to examine the efficacy and hazards of aspirin therapy for a wider range of cardiovascular end points as well as adverse events. We systematically explored for possible sources of heterogeneity and tested for evidence of effect modification using several prespecified study level characteristics. This study also had limitations, several of which were inherent to the meta-analysis. Definitions of some of the clinical outcomes were not consistent across all studies, which could potentially have led to biased estimates. The definition of MACE used in several of the eligible studies included total stroke, which did not differentiate between ischaemic and haemorrhagic stroke events. Hence, this could have resulted in an underestimation of the potential protective effects of aspirin. There appeared to be selective reporting by some studies, as data on some cardiovascular endpoints and adverse events were not reported by some of the included studies. For example, MACE and all-cause mortality outcomes were reported in 10 and 8 trials respectively. We were unable to perform detailed subgroup analyses by clinically relevant subgroups such as appropriate baseline CVD risk groups, appropriate treatment dosages, type of diabetes, and duration of diabetes because of the limited data and inconsistent way of reporting. For example, the dosages and timing of aspirin varied considerably across the studies. These findings should therefore be interpreted with caution given the limitations.

## Conclusions

Aspirin has potential benefits in cardiovascular primary prevention in diabetes, but these may be counterbalanced by an increased bleeding risk. There are suggestions of differential effects of aspirin on cardiovascular outcomes by treatment dosage and duration as well as smoking status, but more data is required. The use of low dose aspirin may need to be individualised and based on each individuals baseline CVD and bleeding risk.

## Additional file

**Additional file 1.** Additional appendices.

## Data Availability

Not applicable.

## Abbreviations

CVD: cardiovascular disease
RCT: randomised controlled trials
MACE: Major Adverse Coronary Events
ADA: American Diabetes Association
ASCEND: A Study of Cardiovascular Events in Diabetes
ASPREE: Aspirin in Reducing Events in the Elderly
GI: gastrointestinal
AHA: American Heart Association
ACCF: American College of Cardiology Foundation
ARRIVE: Aspirin to Reduce Risk of Initial Vascular Events
IPD: Individual Patient Data
MI: myocardial infarction
OR: odds ratio
HR: hazard ratio
RR: relative risk
RRR: relative risk reduction
AR: absolute risk
ARR: absolute risk reduction
NNT: numbers needed to treat
APPRAISE: Aspirin in Primary Prevention of cardiovascular disease in diabetes
ETDRS: The Early Treatment Diabetic Retinopathy Study
PHS: Physicians’ Health Study
PPP: Primary Prevention Project
WHS: Women’s Health Study
