# Supplementary material for: Aspirin has potential benefits for primary prevention of cardiovascular outcomes in diabetes: updated literature-based and individual participant data meta-analyses of randomized controlled trials
Source: Cardiovasc Diabetol. 2019 Jun 3;18:70. doi: 10.1186/s12933-019-0875-4 (PMC6547459; doi:10.1186/s12933-019-0875-4)
Supplement: Supplementary file 1 — Additional file 1. Additional appendices. [file 12933_2019_875_MOESM1_ESM.docx]

**Additional Materials**

| **Appendix S1** | PRISMA checklist |
| --- | --- |
| **Appendix S2** | PRISMA-IPD Checklist |
| **Appendix S3** | MEDLINE literature search strategy |
| **Appendix S4** | Cardiovascular outcomes reported by eligible trials |
| **Appendix S5** | Assessment of risk of bias |
| **Appendix S6** | Relative risks of major adverse cardiovascular events in participants with diabetes for aspirin intervention trials |
| **Appendix S7** | Relative risks of major adverse cardiovascular events in participants with diabetes for aspirin intervention trials on exclusion of the ETDRS trial |
| **Appendix S8** | Relative risks of all-cause mortality in participants with diabetes for aspirin intervention trials |
| **Appendix S9** | Relative risks of myocardial infarction in participants with diabetes for aspirin intervention trials |
| **Appendix S10** | Relative risks of coronary heart disease in participants with diabetes for aspirin intervention trials |
| **Appendix S11** | Relative risks of stroke in participants with diabetes for aspirin intervention trials |
| **Appendix S12** | Relative risks of cardiovascular disease mortality in participants with diabetes for aspirin intervention trials |
| **Appendix S13** | Relative risks of other cardiovascular outcomes in participants with diabetes for aspirin intervention trials |
| **Appendix S14** | Effects of aspirin therapy on major adverse cardiovascular events in participants with diabetes, according to various characteristics |
| **Appendix S15** | Effects of aspirin therapy on all-cause mortality in participants with diabetes, according to various characteristics |
| **Appendix S16** | Effects of aspirin therapy on myocardial infarction in participants with diabetes, according to various characteristics |
| **Appendix S17** | Effects of aspirin therapy on stroke in participants with diabetes, according to various characteristics |
| **Appendix S18** | Effects of aspirin therapy on cardiovascular disease mortality in participants with diabetes, according to various characteristics |
| **Appendix S19** | Assessment of small study effects by funnel plot and Egger’s regression symmetry test |
| **Appendix S20** | Trials contributing individual level data for pooled analysis |
| **Appendix S21** | Effect of aspirin on the primary prevention of major adverse cardiovascular events, myocardial infarction, and stroke, based on pooled analysis of individual level data from two trials using Cox regression analysis |

**Appendix S1.** PRISMA checklist

| **Section/topic** | **Item No** | **Checklist item** | **Reported on page No** |
| --- | --- | --- | --- |
| **Title** | | | |
| Title | 1 | Identify the report as a systematic review, meta-analysis, or both | 1 |
| **Abstract** | | | |
| Structured summary | 2 | Provide a structured summary including, as applicable, background, objectives, data sources, study eligibility criteria, participants, interventions, study appraisal and synthesis methods, results, limitations, conclusions and implications of key findings, systematic review registration number | 2 |
| **Introduction** | | | |
| Rationale | 3 | Describe the rationale for the review in the context of what is already known | 3 |
| Objectives | 4 | Provide an explicit statement of questions being addressed with reference to participants, interventions, comparisons, outcomes, and study design (PICOS) | 4 |
| **Methods** | | | |
| Protocol and registration | 5 | Indicate if a review protocol exists, if and where it can be accessed (such as web address), and, if available, provide registration information including registration number | 4 |
| Eligibility criteria | 6 | Specify study characteristics (such as PICOS, length of follow-up) and report characteristics (such as years considered, language, publication status) used as criteria for eligibility, giving rationale | 4 |
| Information sources | 7 | Describe all information sources (such as databases with dates of coverage, contact with study authors to identify additional studies) in the search and date last searched | 4 |
| Search | 8 | Present full electronic search strategy for at least one database, including any limits used, such that it could be repeated | Appendix 3 |
| Study selection | 9 | State the process for selecting studies (that is, screening, eligibility, included in systematic review, and, if applicable, included in the meta-analysis) | 4-5 |
| Data collection process | 10 | Describe method of data extraction from reports (such as piloted forms, independently, in duplicate) and any processes for obtaining and confirming data from investigators | 5 |
| Data items | 11 | List and define all variables for which data were sought (such as PICOS, funding sources) and any assumptions and simplifications made | 5 |
| Risk of bias in individual studies | 12 | Describe methods used for assessing risk of bias of individual studies (including specification of whether this was done at the study or outcome level), and how this information is to be used in any data synthesis | 5 |
| Summary measures | 13 | State the principal summary measures (such as risk ratio, difference in means). | 5-6 |
| Synthesis of results | 14 | Describe the methods of handling data and combining results of studies, if done, including measures of consistency (such as I^2^ statistic) for each meta-analysis | 5-6 |
| Risk of bias across studies | 15 | Specify any assessment of risk of bias that may affect the cumulative evidence (such as publication bias, selective reporting within studies) | 6 |
| Additional analyses | 16 | Describe methods of additional analyses (such as sensitivity or subgroup analyses, meta-regression), if done, indicating which were pre-specified | 6 |
| **Results** | | | |
| Study selection | 17 | Give numbers of studies screened, assessed for eligibility, and included in the review, with reasons for exclusions at each stage, ideally with a flow diagram | 6 and Figure 1 |
| Study characteristics | 18 | For each study, present characteristics for which data were extracted (such as study size, PICOS, follow-up period) and provide the citations | 6-7, Table 1 |
| Risk of bias within studies | 19 | Present data on risk of bias of each study and, if available, any outcome-level assessment (see item 12). | 7, Appendix 5 |
| Results of individual studies | 20 | For all outcomes considered (benefits or harms), present for each study (a) simple summary data for each intervention group and (b) effect estimates and confidence intervals, ideally with a forest plot | 7-10 |
| Synthesis of results | 21 | Present results of each meta-analysis done, including confidence intervals and measures of consistency | 7-10, Figures 2-5; Appendices 6-12 |
| Risk of bias across studies | 22 | Present results of any assessment of risk of bias across studies (see item 15) | 10, Appendix 5 |
| Additional analysis | 23 | Give results of additional analyses, if done (such as sensitivity or subgroup analyses, meta-regression) (see item 16) | Appendices 14-18 |
| **Discussion** | | | |
| Summary of evidence | 24 | Summarise the main findings including the strength of evidence for each main outcome; consider their relevance to key groups (such as health care providers, users, and policy makers) | 10-11 |
| Limitations | 25 | Discuss limitations at study and outcome level (such as risk of bias), and at review level (such as incomplete retrieval of identified research, reporting bias) | 12-13 |
| Conclusions | 26 | Provide a general interpretation of the results in the context of other evidence, and implications for future research | 13 |
| **Funding** | | | |
| Funding | 27 | Describe sources of funding for the systematic review and other support (such as supply of data) and role of funders for the systematic review | None |

**Appendix S2.** PRISMA-IPD Checklist

| **PRISMA-IPD**  **Section/topic** | **Item No** | **Checklist item** | **Reported on page** |
| --- | --- | --- | --- |
| **Title** | | | |
| Title | 1 | Identify the report as a systematic review and meta-analysis of individual participant data. | 1 |
| **Abstract** | | | |
| Structured summary | 2 | Provide a structured summary including as applicable: | 3 |
|  |  | **Background**: state research question and main objectives, with information on participants, interventions, comparators and outcomes. |  |
|  |  | **Methods**: report eligibility criteria; data sources including dates of last bibliographic search or elicitation, noting that IPD were sought; methods of assessing risk of bias. |  |
|  |  | **Results**: provide number and type of studies and participants identified and number (%) obtained; summary effect estimates for main outcomes (benefits and harms) with confidence intervals and measures of statistical heterogeneity. Describe the direction and size of summary effects in terms meaningful to those who would put findings into practice. |  |
|  |  | **Discussion:** state main strengths and limitations of the evidence, general interpretation of the results and any important implications. |  |
|  |  | **Other:** report primary funding source, registration number and registry name for the systematic review and IPD meta-analysis. |  |
| **Introduction** | | | |
| Rationale | 3 | Describe the rationale for the review in the context of what is already known. | 4-5 |
| Objectives | 4 | Provide an explicit statement of the questions being addressed with reference, as applicable, to participants, interventions, comparisons, outcomes and study design (PICOS). Include any hypotheses that relate to particular types of participant-level subgroups. | 5 |
| **Methods** | | | |
| Protocol and registration | 5 | Indicate if a protocol exists and where it can be accessed. If available, provide registration information including registration number and registry name. Provide publication details, if applicable. | 5 |
| Eligibility criteria | 6 | Specify inclusion and exclusion criteria including those relating to participants, interventions, comparisons, outcomes, study design and characteristics (e.g. years when conducted, required minimum follow-up). Note whether these were applied at the study or individual level i.e. whether eligible participants were included (and ineligible participants excluded) from a study that included a wider population than specified by the review inclusion criteria. The rationale for criteria should be stated. | 6 |
| Identifying studies - information sources | 7 | Describe all methods of identifying published and unpublished studies including, as applicable: which bibliographic databases were searched with dates of coverage; details of any hand searching including of conference proceedings; use of study registers and agency or company databases; contact with the original research team and experts in the field; open adverts and surveys. Give the date of last search or elicitation. | 6 |
| Identifying studies - search | 8 | Present the full electronic search strategy for at least one database, including any limits used, such that it could be repeated. | Appendix 3 |
| Study selection processes | 9 | State the process for determining which studies were eligible for inclusion. | 6 |
| Data collection processes | 10 | Describe how IPD were requested, collected and managed, including any processes for querying and confirming data with investigators. If IPD were not sought from any eligible study, the reason for this should be stated (for each such study). | 6 |
|  |  | If applicable, describe how any studies for which IPD were not available were dealt with. This should include whether, how and what aggregate data were sought or extracted from study reports and publications (such as extracting data independently in duplicate) and any processes for obtaining and confirming these data with investigators. |  |
| Data items | 11 | Describe how the information and variables to be collected were chosen. List and define all study level and participant level data that were sought, including baseline and follow-up information. If applicable, describe methods of standardising or translating variables within the IPD datasets to ensure common scales or measurements across studies. | Appendix 20 |
| IPD integrity | A1 | Describe what aspects of IPD were subject to data checking (such as sequence generation, data consistency and completeness, baseline imbalance) and how this was done. | 6 |
| Risk of bias assessment in individual studies. | 12 | Describe methods used to assess risk of bias in the individual studies and whether this was applied separately for each outcome. If applicable, describe how findings of IPD checking were used to inform the assessment. Report if and how risk of bias assessment was used in any data synthesis. | 7 |
| Specification of outcomes and effect measures | 13 | State all treatment comparisons of interests. State all outcomes addressed and define them in detail. State whether they were pre-specified for the review and, if applicable, whether they were primary/main or secondary/additional outcomes. Give the principal measures of effect (such as risk ratio, hazard ratio, difference in means) used for each outcome. | 6-7 |
| Synthesis methods | 14 | Describe the meta-analysis methods used to synthesise IPD. Specify any statistical methods and models used. Issues should include (but are not restricted to):   - Use of a one-stage or two-stage approach. - How effect estimates were generated separately within each study and combined across studies (where applicable). - Specification of one-stage models (where applicable) including how clustering of patients within studies was accounted for. - Use of fixed or random effects models and any other model assumptions, such as proportional hazards. - How (summary) survival curves were generated (where applicable). - Methods for quantifying statistical heterogeneity (such as I^2^ and τ^2^). - How studies providing IPD and not providing IPD were analysed together (where applicable). - How missing data within the IPD were dealt with (where applicable). | **7** |
| Exploration of variation in effects | A2 | If applicable, describe any methods used to explore variation in effects by study or participant level characteristics (such as estimation of interactions between effect and covariates). State all participant-level characteristics that were analysed as potential effect modifiers, and whether these were pre-specified. | 7 |
| Risk of bias across studies | 15 | Specify any assessment of risk of bias relating to the accumulated body of evidence, including any pertaining to not obtaining IPD for particular studies, outcomes or other variables. | 7 |
| Additional analyses | 16 | Describe methods of any additional analyses, including sensitivity analyses. State which of these were pre-specified. | 7 |
| **Results** | | | |
| Study selection and IPD obtained | 17 | Give numbers of studies screened, assessed for eligibility, and included in the systematic review with reasons for exclusions at each stage. Indicate the number of studies and participants for which IPD were sought and for which IPD were obtained. For those studies where IPD were not available, give the numbers of studies and participants for which aggregate data were available. Report reasons for non-availability of IPD. Include a flow diagram. | 8 |
| Study characteristics | 18 | For each study, present information on key study and participant characteristics (such as description of interventions, numbers of participants, demographic data, unavailability of outcomes, funding source, and if applicable duration of follow-up). Provide (main) citations for each study. Where applicable, also report similar study characteristics for any studies not providing IPD. | Table 1; Appendix 20 |
| IPD integrity | A3 | Report any important issues identified in checking IPD or state that there were none. | None |
| Risk of bias within studies | 19 | Present data on risk of bias assessments. If applicable, describe whether data checking led to the up-weighting or down-weighting of these assessments. Consider how any potential bias impacts on the robustness of meta-analysis conclusions. | Not applicable |
| Results of individual studies | 20 | For each comparison and for each main outcome (benefit or harm), for each individual study report the number of eligible participants for which data were obtained and show simple summary data for each intervention group (including, where applicable, the number of events), effect estimates and confidence intervals. These may be tabulated or included on a forest plot. | Not applicable |
| Results of syntheses | 21 | Present summary effects for each meta-analysis undertaken, including confidence intervals and measures of statistical heterogeneity. State whether the analysis was pre-specified, and report the numbers of studies and participants and, where applicable, the number of events on which it is based. | 9; Figure 5 |
|  |  | When exploring variation in effects due to patient or study characteristics, present summary interaction estimates for each characteristic examined, including confidence intervals and measures of statistical heterogeneity. State whether the analysis was pre-specified. State whether any interaction is consistent across trials. |  |
|  |  | Provide a description of the direction and size of effect in terms meaningful to those who would put findings into practice. |  |
| Risk of bias across studies | 22 | Present results of any assessment of risk of bias relating to the accumulated body of evidence, including any pertaining to the availability and representativeness of available studies, outcomes or other variables. | Not applicable |
| Additional analyses | 23 | Give results of any additional analyses (e.g. sensitivity analyses). If applicable, this should also include any analyses that incorporate aggregate data for studies that do not have IPD. If applicable, summarise the main meta-analysis results following the inclusion or exclusion of studies for which IPD were not available. |  |
| **Discussion** | | | |
| Summary of evidence | 24 | Summarise the main findings, including the strength of evidence for each main outcome. | 11-12 |
| Strengths and limitations | 25 | Discuss any important strengths and limitations of the evidence including the benefits of access to IPD and any limitations arising from IPD that were not available. | 15-16 |
| Conclusions | 26 | Provide a general interpretation of the findings in the context of other evidence. | 12-15 |
| Implications | A4 | Consider relevance to key groups (such as policy makers, service providers and service users). Consider implications for future research. | 13-15 |
| **Funding** | | | |
| Funding | 27 | Describe sources of funding and other support (such as supply of IPD), and the role in the systematic review of those providing such support. | 19 |

**Appendix S3.** MEDLINE literature search strategy

Relevant controlled trials published from November 10, 2015 (date last searched) were identified through electronic searches not limited to the English language using MEDLINE, EMBASE, Web of Science, and Cochrane databases. Electronic searches were supplemented by scanning reference lists of articles identified for all relevant studies (including review articles), and by hand searching of relevant journals. The computer-based searches combined search terms related to (1) the intervention, aspirin (e.g., *aspirin, salicylic acid, and salicylates*) and (2) diabetes (e.g., *diabetes mellitus, type 2 diabetes, and type 1 diabetes*) and primary prevention (e.g., *primary prevention*).

1 exp Aspirin/ (3796)

2 exp Salicylic Acid/ (1067)

3 exp Salicylates/ (5994)

4 exp Diabetes Mellitus/ (61769)

5 exp Diabetes Mellitus, Type 2/ (26911)

6 exp Primary Prevention/ (17375)

7 1 or 2 or 3 (5994)

8 4 or 5 (61769)

9 6 and 7 and 8 (17)

Each part was specifically translated for searching alternative databases.

**Appendix S4.** Cardiovascular outcomes reported by eligible trials

| **Name of study** | **MACE outcome** | **Other outcomes** | **ICD codes and ascertainment** |
| --- | --- | --- | --- |
| BMD | MI, stroke (ischemic, hemorrhagic, unknown), vascular death (including sudden death, pulmonary embolism and hemorrhage) | All-cause mortality, MI, ischemic and hemorrhagic stroke, CHD death, sudden cardiac death | Deaths and non-fatal events reported by the subjects were coded according to ICD-9 codes. All deaths and any non-fatal vascular events were coded by medically qualified staff |
| PHS | CVD mortality, MI and stroke (ischemic, hemorrhagic, unknown) | All-cause mortality, CVD mortality, CHD death, MI, stroke, haemorrhagic and ischaemic stroke | Diagnoses of CVDs or deaths were confirmed by an End Points Committee of physicians, that included two internists, a cardiologist, and a neurologist |
| ETDRS | CVD death, nonfatal MI, and stroke | All-cause mortality, CVD mortality, fatal and nonfatal MI, fatal and nonfatal stroke, sudden coronary death, cerebrovascular death | ICD codes not reported. The Mortality and Morbidity Classification Committee (internists and cardiologists) coded the deaths and hospitalizations for cardiovascular  events |
| TPT | Not reported | All-cause mortality, CHD death, fatal and nonfatal MI, haemorrhagic and ischaemic stroke | Not reported |
| HOT | Fatal and non-fatal MI, fatal and non-fatal stroke (ischemic, hemorrhagic, unknown), ‘all other cardiovascular deaths’ | All-cause mortality, CVD mortality, MI, stroke, CHD | ICD codes not reported. Classification of all reported events was made by the Independent Clinical Event Committee based on all available information |
| PPP | CVD mortality, nonfatal MI and nonfatal stroke (ischemic, hemorrhagic, unknown) | All-cause mortality, CVD mortality, CHD death, MI, stroke, haemorrhagic and ischaemic stroke | Cardiovascular deaths classified according to ICD-9 codes 390-459. Validation of the clinical events was assurred by an ad hoc committee of expert clinicians |
| WHS | CVD mortality, non-fatal MI, non-fatal stroke (ischemic or hemorrhagic) | All-cause mortality, CVD mortality, CHD death, MI, stroke, haemorrhagic and ischaemic stroke | No ICD codes reported. Endpoints were confirmed by an end-points committee of physicians |
| POPADAD | Not reported | All-cause mortality, fatal and nonfatal MI, fatal and nonfatal stroke, CHD death, stroke death, TIA, revascularization, angina | Not reported |
| JPAD | Fatal and nonfatal CHD, fatal and nonfatal cerebrovascular disease | All-cause mortality, fatal and nonfatal MI, fatal and nonfatal stroke, fatal and nonfatal CHD, unstable and stable angina, ischemic and hemorrhagic stroke, TIA | Not reported |
| JPPP | CVD mortality, non-fatal MI, non-fatal stroke (ischemic, hemorrhagic) | All-cause mortality, CVD mortality, MI, ischemic and hemorrhaguc stroke | Not reported |
| ASPREE | CHD death, non-fatal MI, fatal and non-fatal stroke (ischemic, hemorrhagic, uncertain and subarachnoid hemorrhage strokes) | All-cause mortality, stroke or CHD death, MI, ischemic and hemorrhagic stroke, subarachnoid haemorrhage | Endpoints were validated by adjudicators |
| ASCEND | Non-fatal MI, non-fatal stroke (ischemic only) or TIA, vascular death | All-cause mortality, CVD mortality, MI, ischemic stroke | All endpoints were adjudicated centrally by clinicians |

ASCEND, A Study of Cardiovascular Events in Diabetes; ASPREE, Aspirin to Reduce Risk of Initial Vascular Events; BMD, British male doctors; CHD, coronary heart disease; CVD, cardiovascular disease; ETDRS, Early Treatment Diabetic Retinopathy Study; HOT, Hypertension Optimal Treatment; ICD, International Classification of Diseases; IHD, ischaemic heart disease; JPAD, Japanese Primary Prevention of Atherosclerosis with Aspirin for Diabetes; JPPP, Japanese Primary Prevention Project; MACE, major adverse cardiovascular events; MI, myocardial infarction; NR, not reported; PHS, Physicians’ Health Study; POPADAD, Prevention Of Progression of Arterial Disease And Diabetes; PPP, Primary Prevention Project; TIA, transient ischemic attack; WHS, Women’s Health Study

**Appendix S5.** Assessment of risk of bias

ASCEND, A Study of Cardiovascular Events in Diabetes; ASPREE, Aspirin to Reduce Risk of Initial Vascular Events; BMD, British male doctors; ETDRS, Early Treatment Diabetic Retinopathy Study; HOT, Hypertension Optimal Treatment; IHD, ischaemic heart disease; JPAD, Japanese Primary Prevention of Atherosclerosis with Aspirin for Diabetes; JPPP, Japanese Primary Prevention Project; NR, not reported; PHS, Physicians’ Health Study; POPADAD, Prevention Of Progression of Arterial Disease And Diabetes; PPP, Primary Prevention Project; WHS, Women’s Health Study

**Appendix S6.** Relative risks of major adverse cardiovascular events in participants with diabetes for aspirin intervention trials

Study acronyms are provided in **Appendix S5**; CI, confidence interval (bars)

**Appendix S7.** Relative risks of major adverse cardiovascular events in participants with diabetes for aspirin intervention trials on exclusion of the ETDRS trial

Study acronyms are provided in **Appendix S5**; CI, confidence interval (bars)

**Appendix S8.** Relative risks of all-cause mortality in participants with diabetes for aspirin intervention trials

Study acronyms are provided in **Appendix S5**; CI, confidence interval (bars)

**Appendix S9.** Relative risks of myocardial infarction in participants with diabetes for aspirin intervention trials

Study acronyms are provided in **Appendix S5**; CI, confidence interval (bars)

**Appendix S10.** Relative risks of coronary heart disease in participants with diabetes for aspirin intervention trials

Study acronyms are provided in **Appendix S5**; CI, confidence interval (bars)

**Appendix S11.** Relative risks of stroke in participants with diabetes for aspirin intervention trials

Study acronyms are provided in **Appendix S5**; CI, confidence interval (bars)

**Appendix S12.** Relative risks of cardiovascular disease mortality in participants with diabetes for aspirin intervention trials

Study acronyms are provided in **Appendix S5**; CI, confidence interval (bars)

**Appendix S13.** Relative risks of other cardiovascular outcomes in participants with diabetes for aspirin intervention trials

Study acronyms are provided in **Appendix S5**; CHD, coronary heart disease; CI, confidence interval (bars); CVD, cardiovascular disease; MI, myocardial infarction; TIA, transient ischaemic attack

**Appendix S14.** Effects of aspirin therapy on major adverse cardiovascular events in participants with diabetes, according to various characteristics

The summary estimates presented were calculated using random effects models; CI, confidence interval (bars); CVD, cardiovascular disease; *, *P*-value for meta-regression; **, 7 trials reported compliance data

**Appendix S15.** Effects of aspirin therapy on all-cause mortality in participants with diabetes, according to various characteristics

The summary estimates presented were calculated using random effects models; CI, confidence interval (bars); CVD, cardiovascular disease; *, *P*-value for meta-regression; **, 5 trials reported compliance data

**Appendix S16.** Effects of aspirin therapy on myocardial infarction in participants with diabetes, according to various characteristics

The summary estimates presented were calculated using random effects models; CI, confidence interval (bars); CVD, cardiovascular disease; *, *P*-value for meta-regression; **, 4 trials reported compliance data

**Appendix S17.** Effects of aspirin therapy on stroke in participants with diabetes, according to various characteristics

The summary estimates presented were calculated using random effects models; CI, confidence interval (bars); CVD, cardiovascular disease; *, *P*-value for meta-regression; **, 4 trials reported compliance data

**Appendix S18.** Effects of aspirin therapy on cardiovascular mortality in participants with diabetes, according to various characteristics

The summary estimates presented were calculated using random effects models; CI, confidence interval (bars); CVD, cardiovascular disease; *, *P*-value for meta-regression; **, 5 trials reported compliance data

**Appendix S19.** Assessment of small study effects by funnel plot and Egger’s regression symmetry tests

The dotted lines show 95% confidence intervals around the overall summary estimate calculated using a fixed effect model; MACE, major adverse cardiovascular events

**Appendix S20.** Trials contributing individual level data for pooled analysis

| **Name of study** | **Variables contributed** | **Outcomes contributed** |
| --- | --- | --- |
| PHS | Age, body mass index, systolic blood pressure, total cholesterol, aspirin dosage, aspirin treatment duration, smoking status, history of diabetes, history of hypertension, family history of myocardial infarction, treatment for hypertension at baseline, history of chronic kidney disease, and history of rheumatoid arthritis | Major adverse cardiovascular events, all-cause mortality, cardiovascular death, coronary heart disease death, myocardial infarction, stroke, haemorrhagic stroke, and ischaemic stroke |
| WHS | Age, body mass index, systolic blood pressure, total cholesterol, aspirin dosage, aspirin treatment duration, smoking status, history of diabetes, history of hypertension, family history of myocardial infarction, treatment for hypertension at baseline, history of chronic kidney disease, and history of rheumatoid arthritis | Major adverse cardiovascular events, all-cause mortality, cardiovascular death, coronary heart disease death, myocardial infarction, stroke, haemorrhagic stroke, and ischaemic stroke |
| PPP | Age, gender, body mass index, smoking status, history of hypertension, systolic blood pressure, total cholesterol, and history of rheumatoid arthritis | Major adverse cardiovascular events, all-cause mortality, cardiovascular death, coronary heart disease death, myocardial infarction, stroke, haemorrhagic stroke, ischaemic stroke, gastro-intestinal bleeding |

PHS, Physicians’ Health Study; PPP, Primary Prevention Project; WHS, Women’s Health Study

**Appendix S21.** Effect of aspirin on the primary prevention of major adverse cardiovascular events, myocardial infarction, and stroke, based on pooled analysis of individual level data from two trials using Cox regression analysis

CI, confidence interval (bars); HR, hazard ratio; MACE, major adverse cardiovascular events
